# A Novel Role of Interleukin-6 as a Regulatory Factor of Inflammation-Associated Deterioration in Osteoblast Arrangement

**DOI:** 10.3390/ijms21186659

**Published:** 2020-09-11

**Authors:** Aira Matsugaki, Shun Matsumoto, Takayoshi Nakano

**Affiliations:** Division of Materials and Manufacturing Science, Graduate School of Engineering, Osaka University, 2-1 Yamadaoka, Suita, Osaka 565-0871, Japan; matsugaki@mat.eng.osaka-u.ac.jp (A.M.); shun.matsumoto@mat.eng.osaka-u.ac.jp (S.M.)

**Keywords:** macrophage, osteoblast, cell arrangement, bone microstructure, interleukin-6

## Abstract

Inflammatory disorders are associated with bone destruction; that is, deterioration in bone cell activities are under the control of the innate immune system. Macrophages play a central role in innate immunity by switching their polarized phenotype. A disturbed immune system causes aberrance in the ordered bone matrix microarrangement, which is a dominant determinant of bone tissue functionalization. However, the precise relationship between the immune system and bone tissue organization is unknown. In this study, the controlled *in vitro* co-culture assay results showed that M1-polarized macrophages disrupted the osteoblast alignment, which directly modulate the oriented bone matrix organization, by secreting pro-inflammatory cytokines. Notably, interleukin-6 was found to be a key regulator of unidirectional osteoblast alignment. Our results demonstrated that inflammatory diseases triggered bone dysfunction by regulating the molecular interaction between the immune system and bone tissue organization. These findings may contribute to the development of therapeutic targets for inflammatory disorders, including rheumatoid arthritis.

## 1. Introduction

Important bone functions, including providing structural support to the body and inner organs, are dominated by the unique microarrangement of the bone matrix component [1,2,3]. The ordered collagen/apatite microstructure is closely associated with innate and acquired factors that are related to bone metabolism [4,5], the mechanical environment [6,7], or abnormal cancerous cell functions [8,9]. These factors are controlled by mutual activities of bone cells, which include osteoblast–osteoclast coupling [10], cell motility [11,12], and osteocyte-mediated mechanosensing [13]. In particular, osteoblast arrangement is a direct contributor to the oriented construction of the bone matrix [14,15]. Impaired bone cell activities are linked to various bone diseases [16,17]. For example, inflammatory conditions are associated with osteoporosis and increased risk of fractures [18,19]. These inflammatory conditions elicit bone dysfunction by dysregulated cellular activities. The cytokines activated during the inflammatory process exert effects on bone cell activities and further induce bone loss. This is because the inflammatory related cytokines are operative in not only targeting the inflammatory healing process, but also stimulating osteoblast and osteoclast activation [20,21]. Importantly, inflammatory conditions related to the bone healing process or pathogenic stimuli result in the disorganized microstructure of the bone matrix [22], allowing us to hypothesize that the formation of the skeletal microstructure is under the control of the immune system. Moreover, the tumor microenvironment, which is closely related to inflammation, disrupted the osteoblast arrangement and further induced disorganization of the bone matrix microstructure [8].

The innate immune system is strictly regulated by macrophages [23], the specialized cells involved in the immune response. Macrophages play a central role in inflammation and the subsequent tissue rebuilding process by switching their phenotype; M1-polarized macrophages mediate the inflammatory reaction by producing pro-inflammatory cytokines, whereas M2-polarized macrophages participate in tissue repair and remodeling by producing anti-inflammatory cytokines [24]. M2 macrophages can be further divided into subsets, specifically M2a, M2b, M2c, and M2d based on the gene expression profiles [25]. Among them, M2a macrophages play anti-inflammatory, wound-healing functions. A number of cytokines play important roles in enforcing the innate immune system. Among them, interleukin-6 (IL-6) has long been recognized as a key modulator of the pro-inflammatory process, and it has recently been reported to be involved in modulating bone homeostasis in inflammatory diseases; in particular, it has been developed as a therapeutic target for immune disorders, including rheumatoid arthritis [26,27].

The cooperative relationship between bone tissues and the innate immune system is still not fully understood; hence, a novel approach targeting the molecular mechanisms underlying the bone dysfunction controlled under the immune system is imperative. In this study, an original co-culture model, combining osteoblast cultivation on an anisotropic collagen scaffold with the preferential alignment of collagen fibers and controlled macrophage polarization, was established. The collagen matrix plays an important role as a scaffold for cell adhesion during the tissue regeneration process; cellular responses against collagen alignment mirrors the biological functionality. That is, the evaluation of directional responses against scaffold collagen orientation allows us to understand the cellular ability to construct organized tissues or organs [14]. Further, we showed the unexpected association between osteoblast alignment and the immune system; macrophage activation regulated the ordered arrangement of osteoblast via the IL-6 signaling pathway.

## 2. Results

### 2.1. Macrophage Polarization

**Figure 1:**
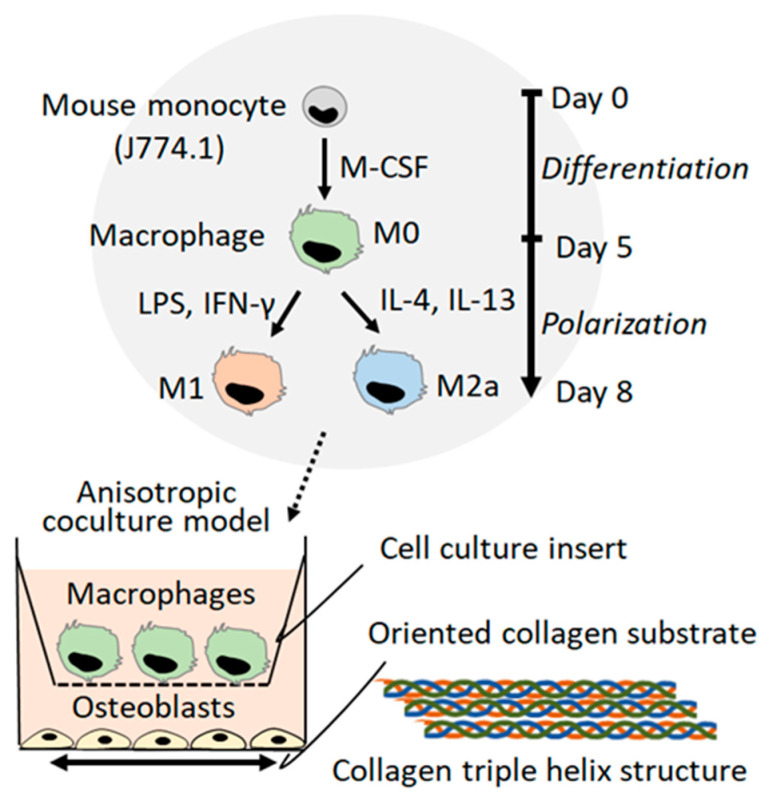
A novel co-culture platform combining the anisotropic cultivation of osteoblasts on oriented collagen substrates and the controlled polarization of macrophages is shown.

**Figure 2:**
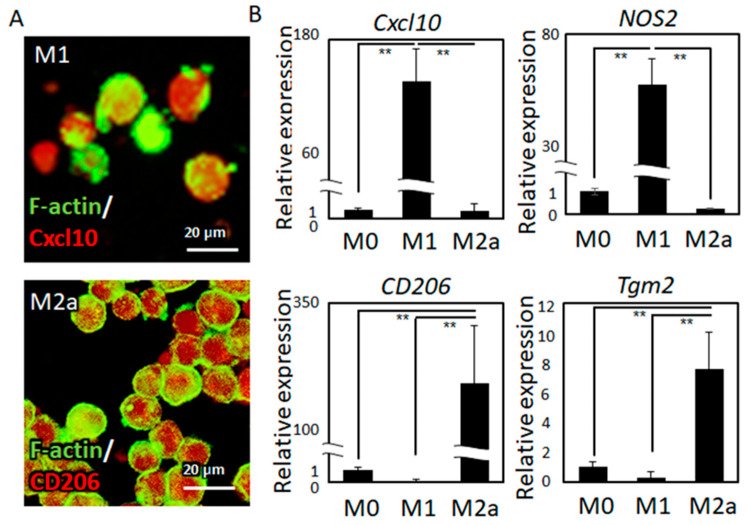
(**A**) Immunocytochemical analysis of Cxcl10 and CD206 marker expression in M1- and M2-subtype macrophages, respectively, is shown. (**B**) Gene expression analysis of *Cxcl10* and *NOS2* as M1 marker genes and *CD206* and *Tgm2* as M2 marker genes is shown; ** *p* < 0.01.

The novel co-culture system of oriented cultivation of osteoblasts and controlled polarization of macrophages was developed (Figure 1). Over five days of differentiation treatment, followed by three days of exposure to polarization stimuli, the monocytes were successfully polarized into M1 and M2a macrophages. Subtype-specific surface protein expression was exhibited by immunocytochemical analysis (Figure 2A).

Relative gene expression analysis revealed that the polarized macrophages showed statistically significant expression of subtype-specific marker genes, including *Cxcl10*, (chemokine (C-X-C motif) ligand 10), *CD206* (cluster of differentiation 206, also known as a mannose receptor), *NOS2* (nitric oxide synthase 2), and *Tgm2* (transglutaminase 2) (Figure 2B).

### 2.2. The Effects of the Macrophage Subtype on Osteoblast Alignment

**Figure 3:**
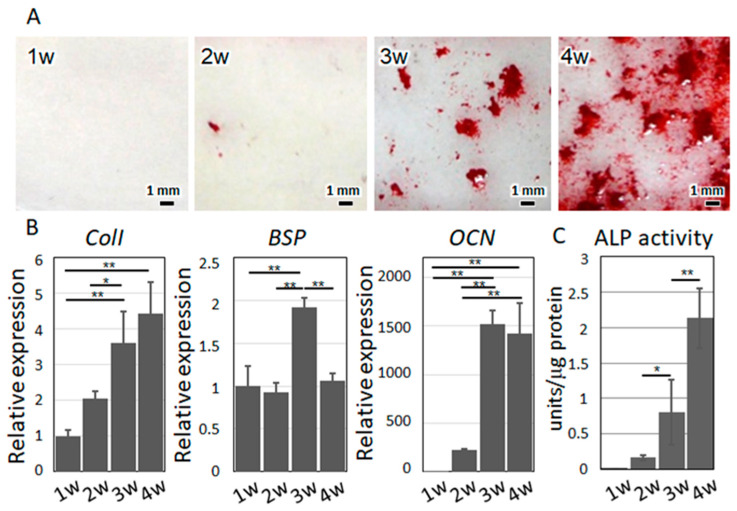
(**A**) Mineralized nodules were visualized by alizarin red S staining. (**B**) Gene expression analysis of osteoblast marker molecules. *ColI*, collagen I; *BSP*, bone sialoprotein; *OCN*, osteocalcin. (**C**) ALP activity depending on the cultivation period is shown. * *p* < 0.05, ** *p* < 0.01.

**Figure 4:**
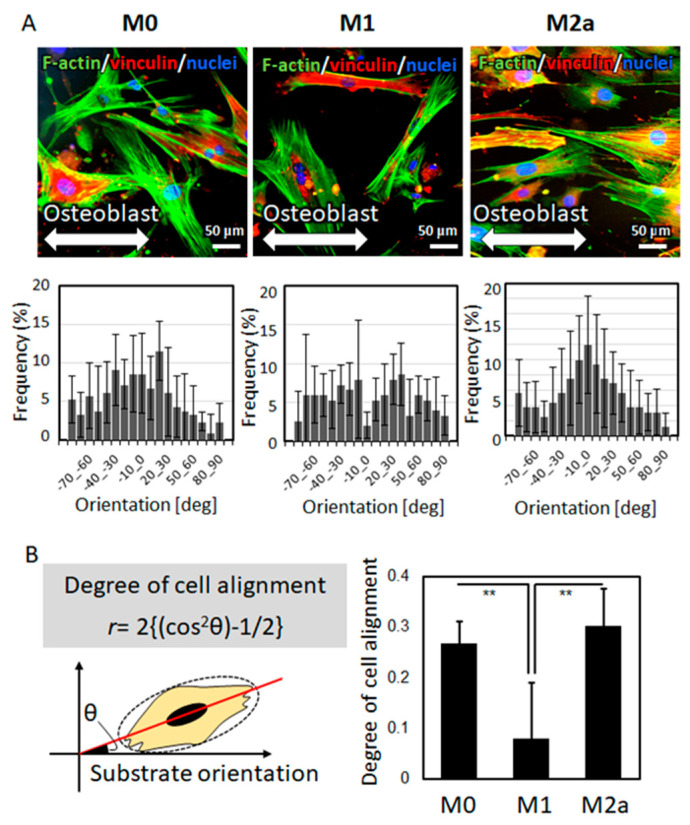
(**A**) Osteoblast alignment, along the orientation of the collagen substrates (indicated by bidirectional arrows), under co-culture with polarized macrophages is shown. The angular cell distribution is shown below the corresponding images. (**B**) Degree of osteoblast alignment was analyzed against the collagen substrate orientation. The comparison of osteoblast alignment with different types of polarized macrophages is shown; ** *p* < 0.01.

The isolated primary cells exhibited characteristics satisfying the osteoblast phenotype. By osteogenetic induction, the cells formed mineralized nodules, with increased alkaline phosphatase (ALP) activity and expression of osteoblast marker genes including collagen I (*ColI*), bone sialoprotein (*BSP*), and osteocalcin (*OCN*) (Figure 3), indicating that the isolated primary cells well reflect the osteoblastic phenotype. Under co-culture with polarized macrophages, the osteoblasts exhibited characteristic responses to the macrophages, depending on the subtype. Under co-culture with M0 or M2a macrophages, the osteoblasts exhibited a high degree of preferential alignment along the orientation of the collagen substrates, whereas under co-culture with M1 macrophages, the osteoblasts showed a significantly lower degree of cell alignment (Figure 4).

### 2.3. The Effects of IL-6 on Osteoblast Alignment

**Figure 5:**
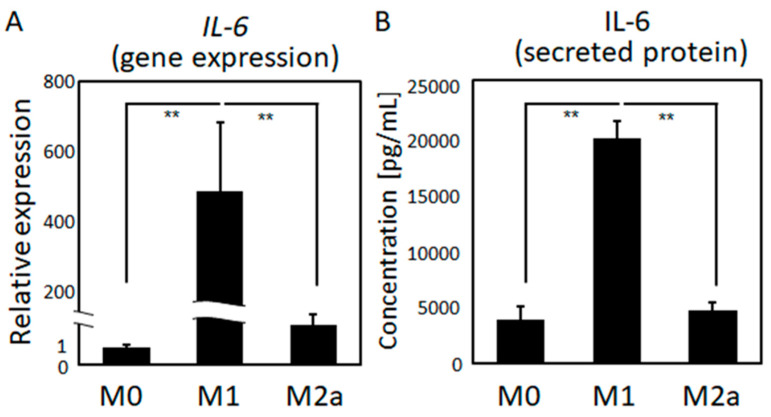
Quantitative comparison of the (**A**) gene expression and (**B**) secreted protein levels of IL-6 among the three types of polarized macrophages is shown; ** *p* < 0.01.

**Figure 6:**
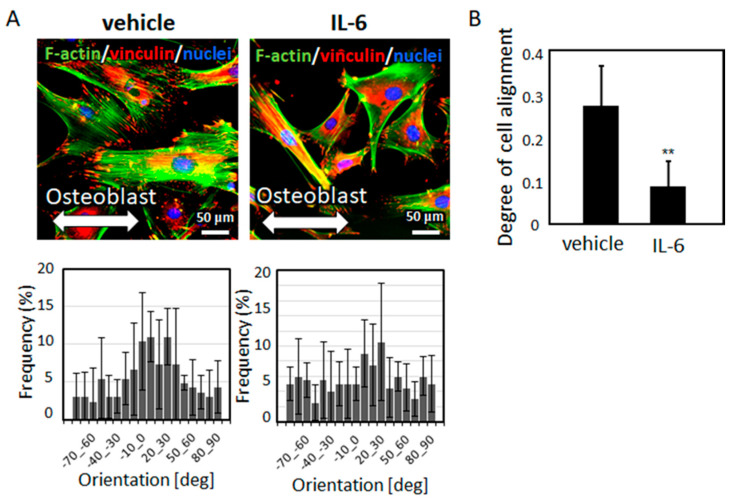
(**A**) Osteoblast alignment, along the orientation of the collagen substrates (indicated by bidirectional arrows), after IL-6 treatment is shown. The angular cell distribution is shown below the corresponding images. (**B**) The degree of cell alignment is compared between the vehicle and IL-6-treated osteoblasts; ** *p* < 0.01, vs. vehicle.

The gene expression and secreted protein levels of IL-6, the major immune system mediator, were significantly higher in M1 macrophages than in quiescent (M0) and M2a macrophages (Figure 5). IL-6 treatment significantly prompted osteoblast disarrangement. Intact osteoblasts exhibited a high degree of preferential alignment along the orientation of the substrates, whereas IL-6-stimulated cells exhibited a lower degree of alignment (Figure 6).

## 3. Discussion

The immune system shares several molecular pathways and cytokines responsible for the regulation of bone integrity [28]. Immunological defects induce abnormal bone phenotypes that accompany deteriorated bone functions. For example, osteopetrosis is caused by genetic defects related to macrophage differentiation; macrophage-colony stimulating factor (M-CSF)-deficient mice showed immunodeficiency and bone dysfunction related to disordered collagen/apatite preferential alignment [29]. Furthermore, inflammation-related tumorigenesis deteriorates the organized bone matrix microstructure [8]. In this study, the intriguing relationship between the immune system and osteoblast arrangement was elucidated for the first time. Importantly, osteoblast arrangement is a determinant factor for bone matrix orientation. Microstructural damage of the bone matrix is closely related to various physiological conditions, including implantation-related inflammation, the bone healing process, and autoimmune disorders. Macrophages play an important role in innate immunity against microbial infections [30], tumor progression [31], and tissue regeneration [32]. Macrophages undergo polarization into M1 or M2 subtypes in response to environmental factors; M1 macrophage activation is associated with inflammation, whereas M2 macrophage activation is related to matrix deposition and remodeling [25]. In this study, an anisotropic co-culture model, providing the directional responses of osteoblasts against polarized macrophages, was developed (Figure 1). M1 polarization was successfully controlled by lipopolysaccharide (LPS) and interferon-γ (IFN-γ), while M2a polarization was successfully controlled by IL-4 and IL-13. *Cxcl10* and *NOS2*, the dominant pro-inflammatory marker genes, were significantly increased in M1 macrophages, whereas *CD206* and *Tgm2*, the typical anti-inflammatory marker molecules, were significantly expressed in M2a macrophages (Figure 2).

Immunological abnormalities or deformities predispose bones to fracture, deteriorating the bone function normally secured by the anisotropic microstructure of the bone matrix [22]. The ordered microarrangement of the bone matrix is constructed by osteoblasts which represent the strictly-controlled unidirectional alignment [14,33]. Osteoblast arrangement is disturbed in pathological conditions, including cancer metastasis [11], resulting in the disordered bone matrix microstructure. Unidirectional osteoblast alignment is strictly controlled by focal adhesion assembly, which determines the directional organization of collagen/apatite [34]. The present study revealed the role of the relationship between macrophage activation and osteoblasts in controlling the anisotropic bone microstructure. Intact osteoblasts exhibited unidirectional alignment, along the orientation of the collagen substrates, via molecular interaction between the amino acid sequence of collagen and integrin receptors [35]. M0 and M2a macrophages exerted no significant effects on the osteoblast arrangement. In contrast, the arrangement of osteoblasts under indirect co-culture with M1 macrophages was significantly disturbed, indicating that pro-inflammatory cytokines altered the cytoskeletal organization and furthered the osteoblast alignment.

Among pro-inflammatory cytokines, IL-6 plays a central role in controlling immune homeostasis and inflammation [26,27]. During the tissue healing process, pro-inflammatory M1 macrophages, compared to M0 and M2a macrophages, secrete abundant levels of IL-6 (Figure 5). It should be noted that M2b macrophages also secrete pro-inflammatory cytokines including IL-6 [25], indicating the possibility of the involvement of M2b macrophages on organized osteoblast arrangement. Moreover, other pro-inflammatory cytokines including TNF-α (tumor necrosis factor-α) and IL-1β (interleukin-1β) can also be involved in control of osteoblast arrangement. Further experiments using conditioned medium in combination with IL-6 neutralizing antibody will be helpful, and it will be reported in our future study. 

**Figure 7:**
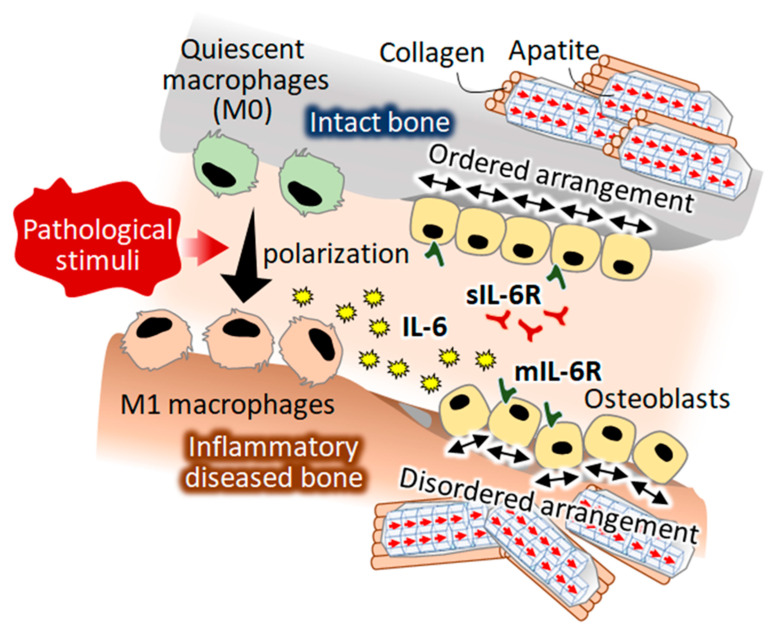
A hypothesized mechanism of pathological stimuli-induced disorganization of bone tissue. A novel function of M1-polarized macrophages in controlling osteoblast arrangement was discovered. Interleukin-6 was found to control osteoblast alignment, which is a direct determinant of the anisotropic bone matrix microarrangement.

In this study, a novel role of IL-6 as a regulatory factor of osteoblast arrangement was discovered (Figure 6). IL-6 treatment of osteoblasts deteriorated the normal arrangement of cells along the orientation of the collagen substrates. IL-6 is recognized as an osteotropic cytokine, which acts through its membrane-associated receptor mIL-6R and soluble receptor sIL-6R; sIL-6R has been reported as being critical for IL-6 signaling in osteoblasts [36]. IL-6 exerts effects on osteoblast differentiation, thereby modulating osteogenesis by regulating the SHP2-related pathways [37]. Both the classical mIL-6R-mediated signaling pathway and the sIL-6R-mediated IL-6 trans-signaling pathway lead to the activation of the JAK/STAT signaling pathway, a major therapeutic target for immunological diseases including rheumatoid arthritis [38,39]. Recently, the IL-6/STAT pathway has been recognized as a regulator of cell adhesion molecules [40,41]. IL-6-stimulated JAK/STAT signaling likely disturbed the focal adhesion organization, resulting in osteoblast disarrangement. The present findings strongly suggested that IL-6 triggered bone dysfunction under inflammatory disease conditions by disrupting the osteoblast arrangement (Figure 7).

## 4. Materials and Methods 

### 4.1. Fabrication of the Oriented Collagen Substrates

The oriented collagen substrates were prepared by the hydrodynamic extrusion method [14]. The deposition of porcine skin collagen type I (10 mg/mL in 0.02 N acetic acid; Nippi, Tokyo, Japan) in phosphate-buffered saline (PBS, 10× concentration) was controlled using a three-axis robotic arm (SM300-3A; Musashi Engineering, Tokyo, Japan), which could regulate the speed and direction of the collagen molecular fibrils. 

### 4.2. Culture of Macrophages

Mouse monocytes J774.1 (1.3 × 10^5^ cells/cm^2^) (RIKEN Cell Bank, Tsukuba, Japan) were cultured in RPMI medium (Nacalai Tesque, Kyoto, Japan) supplemented with 10% heat-inactivated fetal bovine serum (FBS) and penicillin–streptomycin (200 U/mL) for 5 d. M1 or M2 polarization of macrophages was stimulated by culturing the cells with IFN-γ (100 ng/mL; Invitrogen, CA, USA) and LPS (100 ng/mL; Sigma-Aldrich, MO, USA) or IL-4 (40 ng/mL; PeproTech, NJ, USA) and IL-13 (20 ng/mL, PeproTech), with a medium change at day 3.

### 4.3. Osteoblast Isolation and Culture

Primary osteoblasts were isolated from the calvariae of neonatal mice. Calvariae from neonatal C57BL/6 mice were excised under aseptic conditions and placed in ice-cold α-modified Eagle’s medium (α-MEM; GIBCO, Thermo Fisher Scientific, Waltham, MA, USA). The fibrous tissues around the bone were then gently removed. The extracted calvariae tissue were digested with collagenase (Fujifilm Wako, Osaka, Japan)/trypsin (Nacalai Tesque) repeated five times at 37 °C for 15 min each after cutting finely and washing with Hank’s balanced salt solution (HBSS). The obtained supernatants from the third to fifth treatments were collected in α-MEM. The obtained collections were filtered through a cell strainer (BD Biosciences, San Jose, CA, USA), centrifuged, and the supernatant was removed. The obtained filtrates were centrifuged, then the resulting pellets were resuspended in α-MEM containing 10% FBS. Cells were then diluted to 2.0 × 10^4^ cells/mL and seeded into fabricated specimens. For osteogenic induction, the medium was changed twice a week, and after culturing for one week, the medium was supplemented to reach final concentrations of 10 mM β-glycerophosphate (Tokyo Kasei, Tokyo, Japan), 50 μg/mL ascorbic acid (Sigma-Aldrich), and 50 nM dexamethasone (MP Bioscience). All cells were cultured in a humidified incubator (Series 5400; NAPCO, NY, USA) containing 5% CO_2_ and 95% air at 37 °C. All animal experiments were approved by the Osaka University Committee for Animal Experimentation (approval number: 27-2-1, date of approval: 5/11/2016). All experiments were performed in accordance with the related guidelines and regulations for scientific and ethical animal experimentation.

### 4.4. Alizarin Red S Staining

Mineralized nodule formation was detected using alizarin red S staining. Cultured osteoblasts were washed with PBS and fixed in 10% formaldehyde for 20 min. After washing with distilled water, the cells were stained with 1% alizarin red S staining solution (Fujifilm) for 30 min. The excess dye was removed by washing three times with distilled water.

### 4.5. Anisotropic Co-Culture System

A novel anisotropic macrophage-osteoblast co-culture system was established using a combination of cell culture inserts (BD Biosciences), comprised of a polyethylene terephthalate membrane with 1-μm-pores, and artificially controlled oriented collagen substrates. M0, M1, or M2 macrophages were cultured in the upper compartment of the inserts. Primary osteoblasts (1.4 × 10^4^ cells/cm^2^) were cultured on the oriented collagen substrates in the lower compartment of the inserts. 

### 4.6. Fluorescence Imaging 

After culturing for 3 d, the cells were incubated in PBS-0.05% Triton X-100 (PBST) containing 5% normal goat serum (Thermo Fisher Scientific) for 30 min to block the non-specific antibody-binding sites. The cells were incubated with mouse monoclonal antibodies against vinculin (Sigma-Aldrich) at 4 °C for 12 h and then Alexa Fluor 546-conjugated anti mouse IgG (Molecular Probes, Invitrogen, Thermo Fisher Scientific), Alexa Fluor 488-conjugated phalloidin (Molecular Probes, Invitrogen), and Hoechst33342 (Nacalai Tesque). Finally, the cells were washed with PBST and mounted in the Prolong Diamond Antifade reagent (Thermo Fisher Scientific). Fluorescent images were obtained using a fluorescence microscope (BZ-X710; Keyence, Osaka, Japan). 

### 4.7. Quantitative Analysis of Cell Alignment

The orientation of osteoblasts on the oriented collagen substrates, in relation to the running direction of the collagen fibers in the substrate, was examined by taking photographs of the fluorescent-stained cells with magnification (objective lens: 20×) for 30 images per group (n = 5). Cell orientation (θ) was quantitatively analyzed using the Cell Profiler software (Broad Institute, Cambridge, MA, USA) (Figure 2a). The degree of cell orientation was determined using the following equation [11]:

*r* = 2{(cos^2^θ)−1/2} (1)

where the calculated *r* indicates the degree of cell orientation, where *r* = 0 for random orientation and *r* = 1 for completely aligned distribution. For the analysis of the effects of IL-6 treatment on osteoblasts, the cells were treated with IL-6 (1000 ng/mL; R&D Systems, MN, USA). After culturing for 3 d, the cells were fixed and visualized by immunocytochemistry, as described above. The degree of cell alignment was then quantitatively analyzed.

### 4.8. Gene Expression and Protein Secretion Analysis

For gene expression analysis, total RNA was extracted from the macrophages using TRIzol reagent (Thermo Fisher Scientific), according to the manufacturer’s instructions. The quality of extracted RNA was evaluated with absorption ratio of 260/280 nm and 260/230 nm. Gene expression profiling was determined via quantitative reverse transcription-polymerase chain reaction (qRT-PCR) (Step One RT-PCR; Applied Biosystems, CA, USA). Relative change in the gene expression was calculated using the ΔC*t* method, where C*t* is threshold cycle. The expression level was normalized by *Gapdh* as a housekeeping gene. Primers and probes for the targeted genes used for the Taqman Gene Expression Assays were as follows: *Gapdh*, Mm99999915_g1; *ColI*, Mm00801666_g1, *BSP*, Mm00492555_m1, *OCN*, Mm03413826_mH, *Cxcl10*, Mm00445235_m1; *CD206*, Mm01329362_m1; *NOS2*, Mm00440502_m1; *Tgm2*, Mm00436979_m1; *IL-6*, Mm00446190_m1. Alkaline phosphatase (ALP) activity was measured at 7, 14, 21, and 28 days of osteoblast culture using a colorimetric enzymatic assay (Abcam, Cambridge, UK) following the manufacturer’s instructions. Colorimetric intensity at excitation wavelengths of 405 nm was measured using a spectrophotometer (Multiskan Sky, Thermo Fisher Scientific). The measured value was normalized to total protein concentration determined BCA protein assay (Thermo Fisher Scientific). For enzyme-linked immunosorbent assay (ELISA) analysis, the culture medium of macrophages was collected and centrifuged at 2000× *g* for 10 min after culturing for 3 d for stimulation of polarization. IL-6 secretion was measured using the mouse-specific IL-6 ELISA Kit (Abcam, Cambridge, UK), according to the manufacturer’s instructions. 

### 4.9. Statistical Analysis

Statistical significance between two groups was tested using the Student’s *t*-test or Welch’s *t*-test. Tukey’s multiple test was applied for three or more groups. A significance of *p* < 0.05 was required for rejecting the null hypothesis.

## 5. Conclusions

The molecular interactions between the immune system and bone organization remained unsolved. We discovered a novel function of M1-polarized macrophages in controlling osteoblast arrangement. Notably, IL-6 determined the degree of osteoblast alignment. Our findings will open new strategies for developing pharmacological targets for the treatment of non-established microstructural recovery of bone tissue in relation to inflammatory diseases, contributing to the resumption of osteoblast arrangement.
